# The value of coordinated analysis of multimodal atherosclerotic plaque imaging in the assessment of cardiovascular and cerebrovascular events

**DOI:** 10.3389/fcvm.2024.1320222

**Published:** 2024-01-25

**Authors:** Shun Yu, Yonghong Zheng, Xiaomin Dai, Huangjing Chen, Shengsheng Yang, Mingping Ma, Feng Huang, Pengli Zhu

**Affiliations:** ^1^ Shengli Clinical Medical College of Fujian Medical University, Fuzhou, Fujian, Republic of China; ^2^ Department of Radiology, Fujian Provincial Hospital, Fuzhou, Fujian, Republic of China; ^3^ Department of Geriatric Medicine, Fujian Provincial Hospital, Fuzhou, Fujian, Republic of China; ^4^ Fujian Key Laboratory of Geriatrics, Fuzhou, Fujian, Republic of China; ^5^ Fujian Provincial Center for Geriatrics, Fuzhou, Fujian, Republic of China

**Keywords:** atherosclerotic cardiovascular disease (ASCVD), high-resolution vessel wall imaging (HR-VWI), coronary computed tomography angiography (CCTA), stroke, coronary heart disease (CHD)

## Abstract

**Background:**

Although atherosclerosis (AS) can affect multiple vascular beds, previous studies have focused on the analysis of single-site AS plaques.

**Objective:**

The aim of this study is to explore the differences or similarities in the characteristics of atherosclerotic plaque found in the internal carotid artery, cerebral artery, and coronary artery between patients with atherosclerotic cardiovascular disease (ASCVD) and those without events.

**Methods:**

Patients aged ≥ 18 years who underwent both high-resolution vessel wall imaging (HR-VWI) and coronary computed tomography angiography (CCTA) were retrospectively collected and categorized into the ASCVD group and the non-event group. The plaques were then categorized into culprit plaques, non-culprit plaques, and non-event plaques. Plaque morphological data such as stenosis, stenosis grades, plaque length (PL), plaque volume (PV), minimal lumen area (MLA), enhancement grade, and plaque composition data such as calcified plaque volume (CPV), fibrotic plaque volume (FPV), fibro-lipid plaque volume (FLPV), lipid plaque volume (LPV), calcified plaque volume ratio (CPR), fibrotic plaque volume ratio (FPR), fibro-lipid plaque ratio (FLPR), lipid plaque volume ratio (LPR), intraplaque hemorrhage volume (IPHV), and intraplaque hemorrhage volume ratio (IPHR)were recorded and analyzed.

**Results:**

A total of 44 patients (mean age 66 years, SD 9 years, 28 men) were included. In cervicocephalic plaques, the ASCVD group had more severe stenosis grades (*p* = 0.030) and demonstrated significant differences in LPV, LPR, and CPV (*p* = 0.044, 0.030, 0.020) compared with the non-event group. In coronary plaques, the ASCVD group had plaques with greater stenosis (*p* < 0.001), more severe stenosis grades (*p* < 0.001), larger volumes (*p* = 0.001), longer length (*p* = 0.008), larger FLPV (*p* = 0.012), larger FPV (*p* = 0.002), and higher FPR (*p* = 0.043) compared with the non-event group. There were significant differences observed in stenosis (HR-VWI, CCTA: *p* < 0.001, *p* < 0.001), stenosis grades (HR-VWI, CCTA: *p* < 0.001, *p* < 0.001), plaque length (HR-VWI, CCTA: *p* = 0.028, *p* < 0.001), and plaque volume (HR-VWI, CCTA: *p* = 0.013, *p* = 0.018) between the non-event plaque, non-culprit plaque, and culprit plaque. In the image analysis of HR-VWI, there were differences observed between IPHR (*p* < 0.001), LPR (*p* = 0.001), FPV (*p* = 0.011), and CPV (*p* = 0.015) among the three groups of plaques. FLPV and FPV were significantly different among the three different plaque types from the coronary artery (*p* = 0.043, *p* = 0.022).

**Conclusion:**

There is a consistent pattern of change in plaque characteristics between the cervicocephalic and coronary arteries in the same patient.

## 1 Introduction

Atherosclerosis (AS) is a multifactorial and systemic pathophysiologic process that can involve multiple vascular beds such as the aorta, coronary arteries, carotid arteries, and cerebral arteries, leading to the development of atherosclerotic cardiovascular disease (ASCVD). Clinical ASCVD includes acute coronary syndrome (ACS), myocardial infarction (MI), stable or unstable angina, coronary or other revascularization, ischemic stroke, transient ischemic attack, and more. Atherosclerosis within the coronary and cerebral arteries presents a particularly grave threat to individuals, significantly jeopardizing their overall wellbeing and, in severe cases, even leading to fatal outcomes (1).

Despite significant improvements in the prognosis of ASCVD in recent decades, ASCVD remains the leading cause of death and disability worldwide (2). Notably, a study conducted within the CHINA-PAR program, which involved the extended observation of 21,320 Chinese participants over a span exceeding 12 years, revealed an incidence rate of ASCVD at 514.2 cases per 100,000 person-years in men and 293.4 cases per 100,000 person-years in women (3). Cardiovascular and cerebrovascular diseases, such as stroke and ischemic heart disease, are the primary contributors to mortality and the burden of disease among the Chinese population (4).

Current research in ASCVD focuses on ischemic cardiovascular disease, encompassing coronary heart disease (CHD) and stroke. There are many studies focusing on lumen characterization, employing both invasive techniques such as digital subtraction angiography (DSA) and non-invasive “bright blood” angiography techniques, such as magnetic resonance angiography (MRA) and computed tomography angiography (CTA). These studies demonstrate that the degree of stenosis is a very important clinical indicator of ASCVD (5). With the widespread use of coronary computed tomography angiography (CCTA) and high-resolution vessel wall imaging (HR-VWI) of the cervicocephalic, more associations have been found between the characteristics of atherosclerotic plaques and the occurrence of ASCVD. Some atherosclerotic plaques with a mild degree of stenosis (e.g., cerebral stenosis < 50%) may also lead to the development of ASCVD due to some vulnerability features, such as intraplaque hemorrhage (IPH) and higher degree of contrast enhancement (6). Patients with acute chest pain diagnosed with ACS had increased atherosclerotic plaque volume, notably fibro-fatty and necrotic core plaque, when compared with those with stable disease (7).

Previous studies have focused on the analysis of single-site AS plaques and their associated events, whereas the coexistence of AS plaques in coronary, head, and neck arteries is not uncommon in patients with ASCVD. Previous studies have reported that in addition to traditional vascular risk factors, the severity of cervicocephalic artery stenosis and the extent of cervicocephalic atherosclerosis are strongly associated with ≥ 50% asymptomatic CAD (8). Several studies examining the relationship between coronary artery calcification and carotid plaques have indicated that patients with severe coronary artery calcification tend to have more severe carotid artery stenosis (9) and heavier plaque burden (10). Even after adjusting for traditional risk factors, coronary artery calcification still retains the ability to predict cerebrovascular disease (11). Wang et al. (12) demonstrated with invasive imaging that as the carotid artery stenosis becomes more severe, the likelihood of involvement of a greater number of coronary arteries increases. These studies suggest that there are numerous identical risk factors for the development of coronary heart disease and cerebrovascular disease, with a common atherosclerotic basis, and that these conditions often present jointly.

The purpose of this study is to explore the differences or similarities in the characteristics of internal carotid artery, cerebral artery, and coronary atherosclerotic plaques between patients with ASCVD and those without cardiovascular events by using HR-VWI and CCTA imaging techniques.

## 2 Materials and methods

### 2.1 Study population

Between May 2019 and June 2023, we retrospectively enrolled patients who were ≥ 18 years and underwent both HR-VWI and CCTA procedures

The inclusion criteria for the study included the following: (1) HR-VWI and CCTA examinations suggesting the presence of atherosclerotic plaques in the head and neck arteries or coronary arteries; (2) complete clinical history, relevant clinical indicators, and examination data; and (3) quality of coronary CTA and head and neck HR-VWI images for diagnosis and analysis. The study’s exclusion criteria included (1) uncertain diagnosis; (2) patients with previous carotid, cerebral, or coronary artery surgery (e.g., aneurysmal embolization, carotid endarterectomy, or coronary artery bypass grafting); (3) patients who have received endovascular treatment in the head or neck or coronary arteries, such as balloon dilatation and/or stenting; (4) evidence of non-atherosclerotic intracranial vascular disease (e.g., vasculitis, moyamoya disease, dissection, reversible cerebral vasoconstriction syndrome, intracranial tumors, or infectious lesions of the central nervous system); and (5) the interval between VWI and CCTA was more than 1 year.

Clinical information was recorded for each patient, including age, sex, body mass index (BMI), vascular risk factors (the diagnostic criteria are shown in the Online Supplementary Data), history of use of statins, beta-blockers, and other medications, interval between HR-VWI and CCTA examinations, and several blood biochemical indexes.

**Figure 1:**
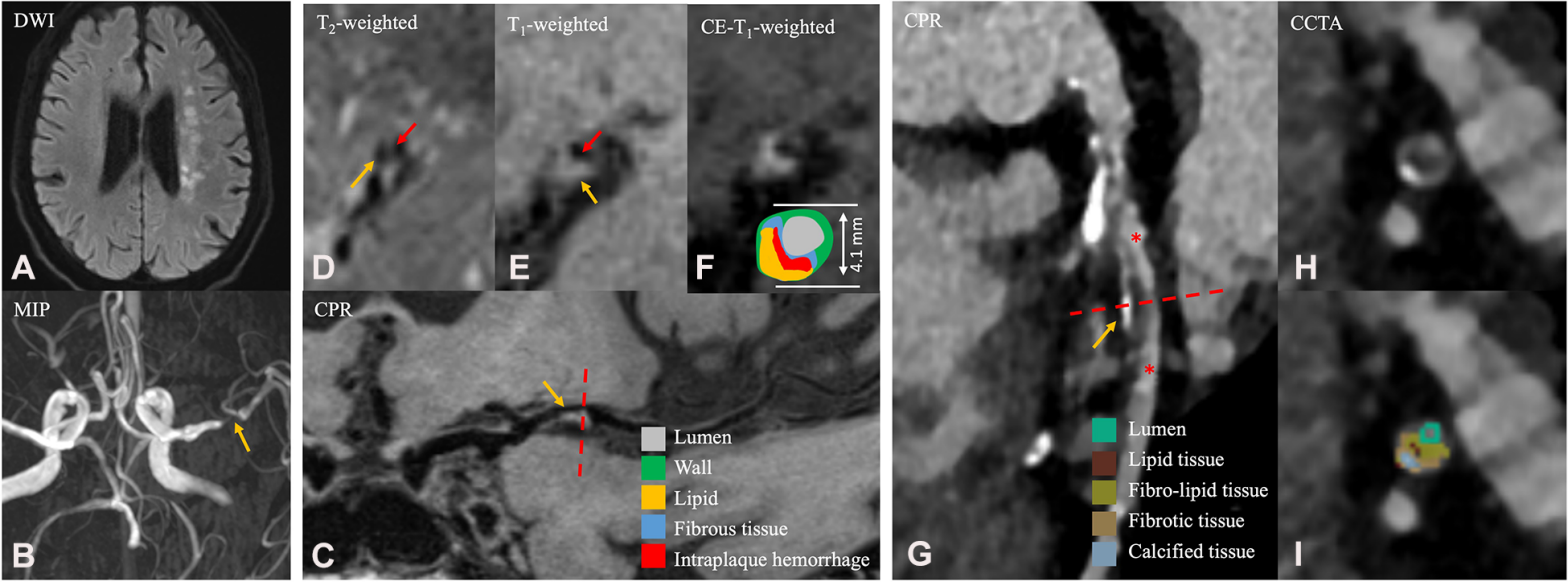
A 65-year-old male patient in the ASCVD group who had a cardiovascular event and a cerebrovascular event. (**A**) Axial diffusion-weighted imaging detects high-signal-intensity lesions in the left radial coronal area. (**B**) Magnetic resonance angiography MIP image shows significant stenosis of the M1 segment of the left MCA. (**C**) CPR images of the M1 segment of left MCA. (**D–F**) Cross-sectional reconstruction of the corresponding plaque in 3D-T2WI, pre- or post-enhancement 3D-T1WI. (**G**) CPR images of the LAD. (**H–I**) Cross-sectional reconstruction and component analysis of the corresponding plaque. A 65-year-old man who presented with dizziness for a week and underwent HR-VWI examination. Diffusion-weighted imaging (**A**) showed multiple acute and subacute cerebral infarctions in the left radial coronal area. These images (**B–F**) exhibit atherosclerotic plaque in the M1 segment of the left MCA (**C**, thin yellow arrow), including thin fibrous cap (**D**, thin red arrow), necrotic core (**D**, thin yellow arrow), IPH (**E**, thin yellow arrow), discontinuity of plaque surface (**E**, thin red arrow), and positive remodeling. Approximately 4 months after the HR-VWI examination, the patient underwent CCTA examination (**G–I**) due to acute chest pain. A segmental, eccentric mixed plaque can be seen in the LAD (**G**, thin yellow arrow). In component analysis (**H**), the main component of plaque is fibro-lipid tissue. These plaques are considered the culprit plaques clinically. MIP, maximum intensity projection; MCA, middle cerebral artery; CPR, curved planar reformation; LAD, left anterior descending branch; IPH, intraplaque hemorrhage; HR-VWI, high-resolution vessel wall imaging; CCTA, coronary computed tomography.

The ASCVD group consisted of patients who experienced a cardiac or/and cerebral ischemic event. The patients with ischemic stroke events met the following criteria: (1) acute ischemic stroke (Figure 1A): a neurological deficit observed upon admission that fitted the definition of acute ischemic stroke, along with a high-intensity signal on diffusion-weighted imaging (DWI) and (2) chronic ischemic stroke: a previous history of ischemic stroke confirmed by hospital records, along with corresponding encephalomalacia in the same territory on fluid attenuated inversion recovery imaging (FLAIR) (13). Patients with cardiac ischemic events included at least any of the following: sudden cardiac death, non-fatal acute myocardial infarction, angina pectoris requiring hospital admission, and the need for coronary revascularization (including percutaneous coronary intervention, thrombolysis, coronary artery bypass grafting). The negative group were patients with atherosclerotic plaques in the head and neck arteries and coronary arteries, but without ischemic cardiovascular events. The research study protocols were approved by the institutional review board, and the need for written informed consent was waived due to the retrospective nature of this investigation.

### 2.2 Imaging examination

#### 2.2.1 MRI examination

All MR images were obtained on a 3T whole-body system (Magnetom Prisma, Siemens) with a 64-channel head and neck coil. The imaging protocol included DWI, T1-weighted and T2-weighted imaging, T2-weighted fluid attenuation inversion recovery imaging (T2-FLAIR), 3D time-of-flight MRA, 3D T1-weighted HR-MRI, 3D T2-weighted HR-MRI, and postcontrast 3D T1-weighted HR-VWI. These imaging techniques were performed using a standardized acquisition method (the parameters are detailed in the Supplementary Table S1). DWI helped the localization of the acute cerebral ischemic lesion (Figure 1A). FLAIR was used to confirm the presence of encephalomalacia.

The HR-VWI procedure was performed using the inversion recovery prepared sampling perfection with application-optimized contrast by using different flip angle evolutions (SPACE) sequence in a sagittal plane optimized for intracranial plaque evaluation and flow signal suppression (14). The SPACE sequence was a non-selective excitation, and the upper boundary reached the A3 segment of the anterior cerebral artery and the inferior boundary included at least 5 cm below the bifurcation of the common carotid artery. Postcontrast 3D T1-weighted HR-VWI was performed with contrast agent injection (0.3 mmol/kg, flush rate, 3 ml/s; 0.9% sodium chloride flush, 20 ml; flush rate, 2 ml/s) using gadobenate dimeglumine.

#### 2.2.2 CCTA examination

Coronary CTA imaging was performed using a third-generation dual-source CT (SOMATOM Force, Siemens Healthineers, Forchheim, Germany). Prior to scanning, patients were instructed to undergo breath-holding training to reduce respiratory motion artifacts. The patient was placed in the supine position with head first, hands raised above the head, and the mid-sagittal plane of the chest perpendicular to the bed. The scanning range extended from the carina of the trachea to 1 cm below the diaphragmatic surface of the heart, left to the apex and to the right border of the heart, including the whole heart.

The scanning mode utilized was prospective electrocardiogram gating stepping. The enhancement procedure was involved injecting 350 mg I/ml of iophorol through the right antecubital vein at a volume of 38–55 ml. The injection rates varied depending on the voltages, and the holding time was 11 s. Subsequently, a 50 ml solution of 0.90% sodium chloride was injected using the same flow rate (the parameters and scanning modes are detailed in the Online Supplemental Data).

### 2.3 Image analysis

All HR-VWI images were independently analyzed by two neuroradiologists with at least 3 months of systematic training using MR-Vascular View (Nanjing Jingsan Medical Science and Technology, Ltd, Jiangsu, China) to analyze plaque in head and neck arteries in accordance with standard methods. The CCTA images were analyzed independently by two independent radiologists using coronary-specific analysis software (CoronaryDoc, ShuKun Technology, Beijing, China). Before analyzing the data, the reviewers were blinded to the clinical data, grouping, and other relevant information of the patients. In case of disagreement between the reviewers, the decision was taken by a third reviewer.

#### 2.3.1 Plaque identification

Due to the small size of the intracranial arteries, only the large arteries were analyzed in HR-VWI, including intracranial internal carotid arteries (ICA), M1 and M2 segments of the middle cerebral arteries (MCA), basilar artery (BA), and intracranial segments of the vertebral arteries (VA). On CCTA, the included vessels were the left anterior descending artery (LAD), left circumflex artery (LCX), and right coronary artery (RCA). A total of 10 vessels were included for analysis, from HR-VWI and CCTA. An atherosclerotic plaque was identified as focal vessel wall thickening with reference to adjacent proximal, distal, or contralateral vessel segments, regardless of whether it caused luminal stenosis.

#### 2.3.2 Plaque classification

In HR-VWI, a plaque was considered a culprit plaque when it was the only lesion within the vascular territory of the stroke. If multiple plaques were present within the same vascular territory of the stroke, the most stenotic lesion would be identified as a culprit plaque (15). In CCTA, culprit lesions associated with cardiac ischemic events were identified based on findings on electrocardiography, wall motion abnormalities on echocardiography, or angiographic appearance during invasive coronary angiography (ICA) (16, 17). In patients in the ASCVD group, all plaques except for the culprit plaque were defined as non-culprit plaques, including all coronary plaques in patients with only cerebral but not cardiac ischemic events, and all head and neck vascular plaques in patients with only cardiac but not cerebral ischemic events. All plaques from the patients in the non-event group were defined as non-event plaques. Multiple plaques could be on the same arterial segment. If a culprit plaque is among them, it will be included. Otherwise, the most stenotic plaque will be included. In the event of disagreement on the definition of plaque, consensus was reached by negotiation.

#### 2.3.3 Plaque morphological characteristics

By means of the multiplanar reformation function of the software, the lumen and vessel boundaries were manually segmented at the most stenotic location of the plaque on the reformatted cross-sectional postcontrast HR-VWI, thereby obtaining the lumen area of plaque (LA_plaque_) and the vessel area of plaque (VA_plaque_). The same method was applied to the normal segments proximal to the plaque to obtain the lumen area of reference (LA_reference_) and the vessel area of reference (VA_reference_). Coronary plaques were analyzed using the same method, which was automated by the software.

The following parameters of culprit plaque, non-culprit plaque, and non-event plaque were derived from the measurements:

$$\mathrm{The}\;\mathrm{degree}\;\mathrm{of}\;\mathrm{stenosis}=\left(1-\frac{LA_{\mathrm{plaque}}}{LA_{\mathrm{reference}}}\right)\times 100\text{\%}$$

The degree of stenosis was measured on black blood 3D-SPACE images using WASID crtieria.

Plaque length (PL) = measuring the length of atherosclerotic plaque on the curved planar reformation (CPR) image.

$$\mathrm{Plaque}\;\mathrm{volume}\;(\mathrm{PV})=\underset{N}{\sum }(VA_{\mathrm{plaque}}\text{-}LA_{\mathrm{plaque}})\times \mathrm{slice}\;\mathrm{thickness}$$

The PV is calculated as shown in the formula above; N is the number of slices.

Minimal lumen area (MLA) = smallest cross-sectional area of the lumen at the location of an atherosclerotic plaque.

Enhancement grade (only in HR-VWI analysis): Grade 0 denotes that the enhancement level is comparable with or lower than that of the intracranial arterial walls without plaque in the same individual. Grade 1 signifies that the enhancement is higher than grade 0 but lower than that of the pituitary infundibulum. Grade 2 indicates that the enhancement is similar to or higher than that of the infundibulum (15).

#### 2.3.4 Plaque compositional characteristics

**Figure 2:**
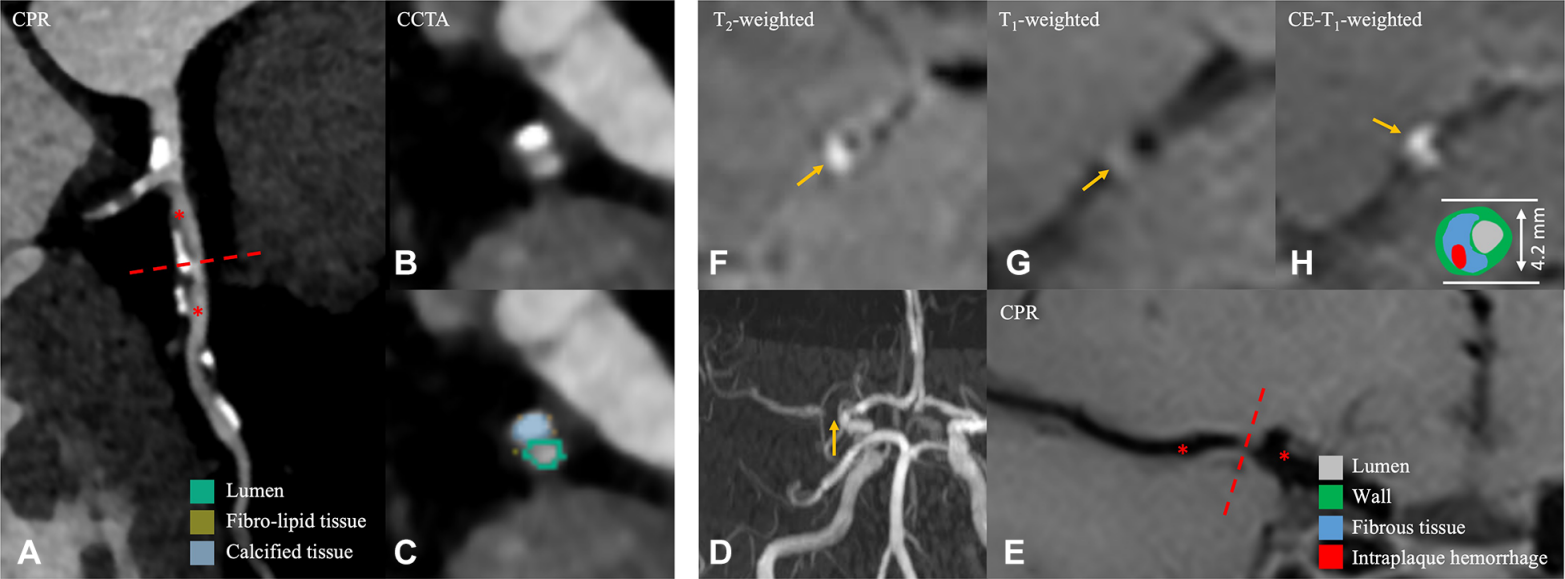
A 56-year-old male patient in the non-event group. (**A**) CPR images of the LAD. (**B,C**) Cross-sectional reconstruction and component analysis of the corresponding plaque. (**D**) Magnetic resonance angiography MIP image shows significant stenosis of the M1 segment of right MCA. (**E**) CPR images of the M1 segment of the right MCA. (**F–H**) Cross-sectional reconstruction of the corresponding plaque in 3D-T2WI, pre- or post-enhancement 3D-T1WI. A 56-year-old man, who was examined for physical examination of CCTA, the most stenotic plaque was located in the LAD coronary artery, and plaque component analysis was predominantly calcified component (**B,C**). Approximately 5 months later, he visited the hospital due to headache for 5 days. HR-VWI was performed and showed no stroke event; the narrowest plaque was located in the M1 segment of the right MCA (**D**, thin yellow arrow), and the component analysis showed that the plaque was mainly composed of fibrous component (**F–H**). CPR, curved planar reformation; LAD, left anterior descending branch; MIP, maximum intensity projection; MCA, middle cerebral artery; HR-VWI, high-resolution vessel wall imaging; CCTA, coronary computed tomography.

In analyzing HR-VWI images (18), a plaque with intraplaque hemorrhage was defined if the signal intensity of its brightest part on the precontrast HR-VWI ≥ 150% of adjacent muscle or pons (Figures 1E, 2G). The IPH volume (IPHV) and IPH volume ratio (IPHR) were calculated in the same way as above. The LRNC of intracranial plaques was isointense/hypointense on T1WI precontrast imaging and hypointense on T2WI imaging (Figure 1D), and LRNC is considered as a lipid component. T2WI high signal adjacent to lumen or enhancement of plaque on postcontrast T1WI is indicated as a fibrous component (Figures 1D, 2F). The calcified component appears as low signal in T2WI and in precontrast or postcontrast T1WI. The lipid plaque volume (LPV) was equal to the volume based on all the pixel area measurements of the lipid plaque category between the proximal and distal ends of the cervicocephalic lesion. Fibrotic plaque volume (FPV), calcified plaque volume (CPV), and IPHV were measured in the same manner as CPV. The lipid plaque volume ratio (LPR) is calculated as LPV divided by the PV. Fibrotic plaque volume ratio (FPR), calcified plaque volume ratio (CPR), and IPHR were calculated in the same way as CPR.

In analyzing CCTA images, the categorization of plaque composition is as follows (Figures 1I, 2C): (1) dense calcium is assigned to HU densities greater than 350 HU; (2) fibrous plaque is assigned to a range of values from 131 to 350 HU; (3) fibro-fatty plaque is assigned to a range of values from 31 to 130 HU; and (4) necrotic core is assigned to a range of values from −30 to 30 HU (19).

The measurements for the analyses of CPV, FPV, fibro-lipid plaque volume (FLPV), LPV, CPR, FPR, fibro-lipid plaque ratio (FLPR), and LPR were obtained using the same methodology as applied to the examined HR-VWI images (7).

### 2.4 Statistical analysis

Categorical variables were presented as frequencies, and continuous variables were presented as means (standard deviations) or median [interquartile range (IQR)]. To compare clinical features of two groups, we used the *t*-test or Wilcoxon rank-sum test (not normally distributed) for continuous data and the *χ*^2^ test for categorical data. Because the same patient might have multiple plaque and considering the inherent variability of arterial atherosclerosis among different patients, a mixed-effects model was employed to analyze multilevel data to assess the differences in plaque characteristics between the two groups (ASCVD group and non-event group) and among three different types of plaques (non-event plaque, non-culprit plaque, and culprit plaque). A two-sided *P*-value of < 0.05 indicated statistical significance in all analyses. Data were analyzed using the SPSS 26.0 (IBM) and R (version 4.2.3) software, and the R package used was lmerTest (20).

## 3 Result

### 3.1 Patient characteristics

We retrospectively reviewed 81 patients who underwent both HR-MRI and CCTA between May 2019 and June 2023. After excluding cases with prior carotid or coronary revascularization (*n* = 5), cases with occlusion of head and neck vessels (*n* = 5), cases with arterial dissection (*n* = 3), cases without atherosclerotic plaque in the coronary vessels (*n* = 13), cases with long interval between CCTA and HR-VWI examination (*n* = 10), and cases with poor image quality (*n* = 1), a total of 44 patients (mean age 66 years, SD 9 years, 28 men) were included in the final analysis.

According to Table 1, out of 44 patients, 24 patients had ASCVD. Among these ASCVD patients, 21 (87.5%) patients experienced a stroke, six (25.0%) patients had CHD, and three (12.5%) patients had both of these ischemic events. Of the patients who had a stroke, 10 patients were in the chronic phase, 11 patients were in the acute phase, and two patients had more than one stroke.

**Table 1:** Baseline characteristics of the study patients.

Variables	All Patients	Non-event group	ASCVD group	*p*
	*N* = 44	*N* = 20	*N* = 24	
Age, mean (SD)	66.14 (9.35)	64.95 (8.31)	67.12 (10.21)	0.449
Sex (male), NO. (%)	28 (63.6)	10 (50.0)	18 (75.0)	0.120
BMI, median [IQR]	23.94 [22.75, 24.75]	24.05 [22.91, 24.75]	23.86 [22.48, 24.82]	0.629
Examination findings				
Systolic BP, mean (SD), mmHg	135.52 (17.20)	139.45 (20.01)	132.25 (14.07)	0.170
Diastolic BP, median [IQR], mmHg	75.00 [71.00, 79.00]	74.50 [71.00, 78.25]	75.00 [70.75, 79.00]	0.915
FBG, median [IQR], mg/dl	5.85 [5.12, 7.60]	5.85 [5.29, 6.85]	5.90 [5.09, 8.62]	0.532
HBA1c, median [IQR], %	6.40 [5.80, 7.45]	6.30 [5.70, 7.12]	6.55 [5.88, 7.80]	0.288
Total cholesterol, mean (SD), mg/dl	4.08 (1.34)	4.29 (1.62)	3.91 (1.05)	0.353
TG, median [IQR], mg/dl	1.32 [0.98, 2.00]	1.23 [0.96, 1.52]	1.53 [1.23, 2.12]	0.150
HDL-C, median [IQR], mg/dl	0.94 [0.78, 1.19]	0.95 [0.84, 1.40]	0.94 [0.74, 1.09]	0.283
LDL-C, mean (SD), mg/dl	2.56 (1.03)	2.56 (1.18)	2.56 (0.91)	0.995
BNP, median [IQR], Pg/ml	115.30 [45.54, 295.30]	106.25 [27.74, 237.85]	132.15 [86.23, 424.50]	0.141
CREA, mean (SD), mmol/L	73.93 (15.61)	69.25 (16.04)	77.83 (14.44)	0.069
eGFR, median [IQR]	89.04 [76.65, 97.68]	94.94 [78.76, 100.45]	84.80 [76.65, 91.90]	0.099
Medical history, No. (%)				
Hypertension	33 (75.0)	13 (65.0)	20 (83.3)	0.185
Diabetes mellitus	21 (47.7)	9 (45.0)	12 (50.0)	0.771
History of smoking	16 (36.4)	4 (20.0)	12 (50.0)	0.060
History of alcohol intake	17 (38.6)	7 (35.0)	10 (41.7)	0.760
Dyslipidemia	34 (77.3)	15 (75.0)	19 (79.2)	1.000
History of antiplatelet drug use	30 (68.2)	12 (60.0)	18 (75.0)	0.342
History of ACEI or ARB use	17 (38.6)	6 (30.0)	11 (45.8)	0.359
History of beta-blocker use	10 (22.7)	5 (25.0)	5 (20.8)	1.000
History of statin use	36 (81.8)	15 (75.0)	21 (87.5)	0.436
Time between CCTA and HR-VWI examination, mean (SD), days	−53.16 (147.15)	−54.50 (150.27)	−52.04 (147.75)	0.957

BP, blood pressure; FBG, fasting blood glucose; HBA1c, hemoglobin A1c; TG, triglyceride; HDL-C, high-density lipoprotein cholesterol; LDL-C, low-density lipoprotein cholesterol; BNP, B-type natriuretic peptide; CREA, creatinine; eGFR, estimated glomerular filtration rate; ACEI, angiotensin converting enzyme inhibitor; ARB, angiotensin receptor blocker; CCTA, coronary computed tomography angiography; HR-VWI, high-resolution vessel wall imaging.

### 3.2 Plaque characteristics in HR-VWI or CCTA between the ASCVD group and non-event group

The imaging features from HR-VWI of the two groups are shown in Table 2. The ASCVD group had 135 plaques, and the non-event group had 98 plaques. The ASCVD group had plaques with more severe stenosis grades (*p* = 0.030). No significant difference in stenosis (*p* = 0.050) and minimum LA (*p* = 0.937) were observed. Compared with the non-event group, the ASCVD group showed significantly more enhanced plaques (*n*, %: 105, 77.8% vs. 42, 42.9%; *p* = 0.006) and a higher grade of plaque enhancement (*p* = 0.001) (Supplementary Table S2).

**Table 2:** Plaque characteristics in HR-VWI between the ASCVD group and non-event group.

Variables		All patients	Non-event group	ASCVD group	*p*
		*N* = 233	*N* = 98	*N* = 135	
Plaque morphological characteristics					
Stenosis, median [IQR]		0.28 (0.22, 0.39)	0.27 (0.20, 0.37)	0.30 (0.24, 0.41)	0.050
Stenosis grades, NO. (%)	Mild	209 (89.7)	93 (94.9)	116 (85.9)	0.030
	Moderate	14 (6.0)	4 (4.1)	10 (7.4)	
	Severe	10 (4.3)	1 (1.0)	9 (6.7)	
Minimum LA, median [IQR], mm^2^		6.10 [3.95, 10.81]	6.50 [4.42, 10.69]	5.73 [3.73, 10.88]	0.937
Plaque length, median [IQR], mm		16.50 [10.80, 26.50]	16.00 [10.00, 24.38]	18.00 [12.00, 29.25]	0.095
Plaque volume, median [IQR], mm^3^		171.58 [99.15, 364.15]	160.62 [101.99, 332.03]	179.01 [96.03, 411.61]	0.108
Plaque compositional characteristics					
IPH NO. (%)		66 (28.3)	25 (25.5)	41 (30.4)	0.387
IPHV, median [IQR], mm^3^		0.00 [0.00, 3.37]	0.00 [0.00, 0.31]	0.00 [0.00, 3.88]	0.161
IPHR, median [IQR]		0.00 [0.00, 0.02]	0.00 [0.00, 0.00]	0.00 [0.00, 0.03]	0.407
LPV, median [IQR], mm^3^		0.00 [0.00, 0.00]	0.00 [0.00, 0.00]	0.00 [0.00, 0.00]	0.044
LPR, median [IQR]		0.00 [0.00, 0.00]	0.00 [0.00, 0.00]	0.00 [0.00, 0.00]	0.030
FPV, median [IQR], mm^3^		51.68 [26.20, 111.15]	48.26 [31.96, 107.97]	55.84 [22.94, 122.78]	0.123
FPR, mean (SD)		0.31 (0.11)	0.31 (0.10)	0.31 (0.12)	0.997
CPV, median [IQR], mm^3^		0.00 [0.00, 0.00]	0.00 [0.00, 0.00]	0.00 [0.00, 0.00]	0.020
CPR, median [IQR]		0.00 [0.00, 0.00]	0.00 [0.00, 0.00]	0.00 [0.00, 0.00]	0.128
Vessel wall, median [IQR], mm^3^		109.05 [59.90, 240.38]	95.82 [60.52, 234.32]	113.98 [59.62, 251.61]	0.203
Vessel wall ratio, mean (SD)		0.65 (0.12)	0.66 (0.10)	0.65 (0.13)	0.535

LA, luminal area; IPHV, IPH volume; IPHR, IPH volume ratio; LPV, lipid plaque volume; LPR, lipid plaque volume ratio; FPV, fibrotic plaque volume; FPR, fibrotic plaque volume ratio; CPV, calcified plaque volume; CPR, calcified plaque volume ratio.

In CCTA imaging features shown in Table 3, the ASCVD group had plaques with greater stenosis (*p* < 0.001), more severe stenosis grades (*p* < 0.001), larger volumes (*p* = 0.001), and longer length (*p* = 0.008) compared with the non-event group.

**Table 3:** Plaque characteristics in CCTA between the ASCVD group and non-event group.

Variable		All patients	Non-event group	ASCVD group	*p*
		*N* = 85	*N* = 33	*N* = 52	
Plaque morphological characteristics					
Stenosis, mean (SD)		0.49 (0.23)	0.37 (0.20)	0.57 (0.21)	<0.001
Stenosis grades, NO. (%)	Mild	42 (49.4)	26 (78.8)	16 (30.8)	<0.001
	Moderate	28 (32.9)	6 (18.2)	22 (42.3)	
	Severe	15 (17.6)	1 (3.0)	14 (26.9)	
Plaque length, median [IQR], mm^2^		6.72 [4.40, 10.25]	4.79 [3.80, 8.46]	7.56 [5.63, 11.76]	0.008
Plaque volume, median [IQR], mm^3^		10.59 [5.53, 31.46]	5.53 [1.43, 18.92]	16.62 [8.09, 40.34]	0.001
Minimum LA, median [IQR], mm^2^		1.97 [0.97, 3.08]	1.34 [0.72, 2.92]	2.13 [1.06, 3.10]	0.182
Plaque compositional characteristics					
LPV, median [IQR], mm^3^		1.01 [0.13, 3.81]	0.84 [0.00, 3.27]	1.33 [0.39, 4.21]	0.197
LPR, median [IQR]		0.10 [0.01, 0.17]	0.09 [0.00, 0.19]	0.10 [0.04, 0.15]	0.697
FLPV, median [IQR], mm^3^		3.60 [1.14, 7.89]	1.73 [0.00, 6.81]	4.78 [2.65, 9.25]	0.012
FLPR, median [IQR]		0.30 [0.20, 0.40]	0.28 [0.00, 0.39]	0.32 [0.24, 0.40]	0.153
FPV, median [IQR], mm^3^		3.40 [1.31, 8.94]	1.46 [0.00, 6.00]	4.77 [2.23, 10.84]	0.002
FPR, median [IQR]		0.33 [0.10, 0.46]	0.27 [0.00, 0.45]	0.35 [0.24, 0.46]	0.043
CPV, median [IQR], mm^3^		1.24 [0.11, 5.14]	0.53 [0.00, 3.35]	1.41 [0.48, 6.16]	0.054
CPR median [IQR]		0.11 [0.03, 0.31]	0.19 [0.00, 1.00]	0.10 [0.04, 0.28]	0.657

LA, luminal area; LPV, lipid plaque volume; LPR, lipid plaque volume ratio; FLPV, fibro-lipid plaque volume; FLPR, fibro-lipid plaque ratio; FPV, fibrotic plaque volume; FPR, fibrotic plaque volume ratio; CPV, calcified plaque volume; CPR, calcified plaque volume ratio.

Regarding plaque components, there were significant differences found in LPV, LPR, and CPV (*p* = 0.044, 0.030, 0.020) when analyzing HR-VWI images. However, the median and interquartile range are unable to delineate the distinctions between the two groups. In CCTA image analysis, the ASCVD group showed significantly large FLPV [median, IQR: 4.78 (2.65, 9.25) vs. 1.73 (0.00, 6.81); *p* = 0.012], large FPV [median, IQR: 4.77 (2.23, 10.84) vs. 1.46 (0.00, 6.00); *p* = 0.002], and higher FPR [median, IQR: 0.35 (0.24, 0.46) vs. 0.27 (0.00, 0.45), *p* = 0.043] compared with the non-event group.

### 3.3 Plaque characteristics in HR-VWI or CCTA between different plaque types

As shown in Tables 4, 5, when analyzing CCTA and HR-VWI images, there were significant differences observed in stenosis (HR-VWI, CCTA: *p* < 0.001, *p* < 0.001), stenosis grades (HR-VWI, CCTA: *p* < 0.001, *p* < 0.001), plaque length (HR-VWI, CCTA: *p* = 0.028, *p* < 0.001), and plaque volume (HR-VWI, CCTA: *p* = 0.013, *p* = 0.018) between the non-event plaque, non-culprit plaque, and culprit plaque. However, the differences in minimum LA (*p* < 0.001) were only observed in head and neck plaques. In terms of head and neck plaque enhancement (Supplementary Table S3), there were significant differences observed among the three groups of plaques (*p* = 0.001), and all culprit plaques were enhanced (*n*, %: 24, 100%). In terms of enhancement grade, there were also significant differences observed among the three groups (*p* < 0.001), and significant differences were also seen between the two subgroups in terms of the culprit plaque and non-culprit plaque, as well as between the culprit plaque and non-event plaque.

**Table 4:** Plaque characteristics in HR-VWI between different plaque types.

Variable		Overall	Non-event plaque	Non-culprit plaque	Culprit plaque	*p*-value
		*n* = 233	*n* = 98	*n* = 111	*n* = 24	
Plaque morphological characteristics						
Stenosis, median [IQR]		0.28[0.22, 0.39]	0.27[0.20, 0.37]	0.29[0.23, 0.40]	0.33[0.26, 0.79]	<0.001*,**
Stenosis grades, NO. (%)	Mild	209 (89.7)	93 (94.9)	101 (91.0)	15 (62.5)	<0.001*,**
	Moderate	14 (6.0)	4 (4.1)	9 (8.1)	1 (4.2)	
	Severe	10 (4.3)	1 (1.0)	1 (0.9)	8 (33.3)	
Minimum LA, median [IQR], mm^2^		6.10 [3.95, 10.81]	6.50 [4.42, 10.69]	6.34 [4.01, 12.01]	4.05 [1.34, 5.58]	<0.001*,**
Plaque length, median [IQR], mm		16.50 [10.80, 26.50]	16.00 [10.00, 24.38]	20.00 [12.00, 30.00]	15.00 [11.70, 25.38]	0.028
Plaque volume, median [IQR], mm^3^		171.58 [99.15, 364.15]	160.62 [101.99, 332.03]	193.80 [110.35, 465.40]	139.07 [86.56, 204.22]	0.013*
Plaque compositional characteristics						
IPH NO. (%)		66 (28.3)	25 (25.5)	28 (25.2)	13 (54.2)	0.004*,**
IPHV, median [IQR], mm^3^		0.00 [0.00, 3.37]	0.00 [0.00, 0.31]	0.00 [0.00, 0.31]	3.73 [0.00, 15.88]	0.371
IPHR, median [IQR]		0.00 [0.00, 0.02]	0.00 [0.00, 0.00]	0.00 [0.00, 0.00]	0.04 [0.00, 0.10]	<0.001*,**
LPV, median [IQR], mm^3^		0.00 [0.00, 0.00]	0.00 [0.00, 0.00]	0.00 [0.00, 0.00]	0.00 [0.00, 1.76]	0.131
LPR, median [IQR]		0.00 [0.00, 0.00]	0.00 [0.00, 0.00]	0.00 [0.00, 0.00]	0.00 [0.00, 0.02]	0.001*,**
FPV, median [IQR], mm^3^		51.68 [26.20, 111.15]	48.26 [31.96, 107.97]	62.00 [27.70, 143.68]	37.55 [19.89, 58.46]	0.011*
FPR, median [IQR]		0.32 [0.24, 0.39]	0.32 [0.25, 0.38]	0.32 [0.23, 0.40]	0.30 [0.24, 0.37]	0.239
CPV, median [IQR], mm^3^		0.00 [0.00, 0.00]	0.00 [0.00, 0.00]	0.00 [0.00, 0.00]	0.00 [0.00, 0.00]	0.015
CPR, median [IQR]		0.00 [0.00, 0.00]	0.00 [0.00, 0.00]	0.00 [0.00, 0.00]	0.00 [0.00, 0.00]	0.162
Vessel wall, median [IQR], mm^3^		109.05 [59.90, 240.38]	95.82 [60.52, 234.32]	130.29 [66.12, 288.07]	83.13 [51.50, 115.94]	0.017*,**
Vessel wall ratio, mean (SD)		0.65 (0.12)	0.66 (0.10)	0.65 (0.12)	0.62 (0.17)	0.22

LA, luminal area; IPHV, IPH volume; IPHR, IPH volume ratio; LPV, lipid plaque volume; LPR, lipid plaque volume ratio; FPV, fibrotic plaque volume; FPR, fibrotic plaque volume ratio; CPV, calcified plaque volume; CPR, calcified plaque volume ratio.

* Significant difference between the culprit plaque and non-culprit plaque.

** Significant difference between the culprit plaque and non-event plaque.

**Table 5:** Plaque characteristics in CCTA between different plaque types.

Variables		Overall	Non-event plaque	Non-culprit plaque	Culprit plaque	*p*-value
		*n* = 85	*n* = 33	*n* = 46	*n* = 6	
Plaque morphological characteristics						
Stenosis, mean (SD), %		0.49 (0.23)	0.37 (0.20)	0.57 (0.21)	0.61 (0.28)	0.001**
Stenosis grades, NO. (%)	Mild	42 (49.4)	26 (78.8)	14 (30.4)	2 (33.3)	<0.001**
	Moderate	28 (32.9)	6 (18.2)	20 (43.5)	2 (33.3)	
	Severe	15 (17.6)	1 (3.0)	12 (26.1)	2 (33.3)	
Plaque length, median [IQR], mm^2^		6.72 [4.40, 10.25]	4.79 [3.80, 8.46]	7.28 [5.26, 11.38]	11.40 [7.81, 24.39]	<0.001*,**
Plaque volume, median [IQR], mm^3^		10.59 [5.53, 31.46]	5.53 [1.43, 18.92]	13.80 [7.38, 31.84]	34.02 [17.29, 66.28]	0.018*,**
Minimum LA, median [IQR], mm^2^		1.97 [0.97, 3.08]	1.34 [0.72, 2.92]	2.13 [1.06, 3.06]	2.19 [1.26, 2.92]	0.403
Plaque compositional characteristics						
LPV, median [IQR], mm^3^		1.01 [0.13, 3.81]	0.84 [0.00, 3.27]	1.13 [0.36, 3.50]	4.93 [0.92, 8.54]	0.436
LPR, median [IQR]		0.10 [0.01, 0.17]	0.09 [0.00, 0.19]	0.10 [0.03, 0.16]	0.08 [0.06, 0.12]	0.902
FLPV, median [IQR], mm^3^		3.60 [1.14, 7.89]	1.73 [0.00, 6.81]	4.78 [2.46, 8.58]	5.94 [4.10, 10.64]	0.043**
FLPR, median [IQR]		0.30 [0.20, 0.40]	0.28 [0.00, 0.39]	0.32 [0.27, 0.40]	0.29 [0.18, 0.38]	0.224
FPV, median [IQR], mm^3^		3.40 [1.31, 8.94]	1.46 [0.00, 6.00]	4.53 [2.35, 10.15]	7.74 [3.39, 25.50]	0.022**
FPR, median [IQR]		0.33 [0.10, 0.46]	0.27 [0.00, 0.45]	0.35 [0.25, 0.46]	0.42 [0.20, 0.44]	0.119
CPV, median [IQR], mm^3^		1.24 [0.11, 5.14]	0.53 [0.00, 3.35]	1.37 [0.39, 3.37]	8.14 [1.18, 18.58]	0.163
CPR median [IQR]		0.11 [0.03, 0.31]	0.19 [0.00, 1.00]	0.09 [0.04, 0.28]	0.11 [0.07, 0.24]	0.21

LA, luminal area; LPV, lipid plaque volume; LPR, lipid plaque volume ratio; FLPV, fibro-lipid plaque volume; FLPR, fibro-lipid plaque ratio; FPV, fibrotic plaque volume; FPR, fibrotic plaque volume ratio; CPV, calcified plaque volume; CPR, calcified plaque volume ratio.

* Significant difference between the culprit plaque and non-culprit plaque.

** Significant difference between the culprit plaque and non-event plaque.

In the image analysis of HR-VWI (Table 4) regarding plaque components, there were differences observed between IPHR (*p* < 0.001), LPR (*p* = 0.001), FPV (*p* = 0.011), and CPV (0.015) among the three groups of plaques. In the subgroup analysis, IPHR and LPR differed between the culprit and non-event plaques [median, IQR: 0.04 (0.00, 0.10) vs. 0.00 (0.00, 0.00), *p* < 0.001; 0.00 (0.00, 0.02) vs. 0.00 (0.00, 0.00), *p* = 0.001], while IPHR, LPR, and FPR differed between the culprit and non-culprit plaques [median, IQR: 0.04 (0.00, 0.10) vs. 0.00 (0.00, 0.00), *p* < 0.001; 0.00 (0.00, 0.02) vs. 0.00 (0.00, 0.00), *p* = 0.004; 37.55 (19.89, 58.46) vs. 62.00 (27.70, 143.68), *p* = 0.019]. However, CPV showed no difference in the subgroup analysis. There were also significant differences between the number of plaques showing IPH among the three groups of plaques (*p* = 0.004) and also differences between the two subgroups: culprit plaque and non-culprit plaque (n, %: 13 54.2% vs. 28 25.2%, *p* = 0.002) and culprit plaque and non-event plaque (n, %: 13 54.2% vs. 25 25.5%, *p* = 0.013).

In CCTA imaging features (Table 5), FLPV and FPV were significantly different among the three different plaque types (*p* = 0.043, *p* = 0.022). In the subgroup analysis, in FLPV, there was a statistical difference observed between the culprit plaque and non-event plaque [median, IQR: 5.94 (4.10, 10.64) vs. 1.73 (0.00, 6.81), *p* = 0.029], but no difference was observed between the non-culprit plaque and culprit plaque (*p* = 0.116). FPV yields similar results to those of FLPV, with the exception of the difference between the culprit plaque and non-event plaque [median, IQR: 7.74 (3.39, 25.50) vs. 1.46 (0.00, 6.00), *p* = 0.016].

## 4 Discussion

Atherosclerosis is a systemic disease that involves multiple vascular beds. Individuals who are prone to developing adverse plaque characteristics often manifest a higher number of such lesions in various locations and across different vascular beds over time. Consequently, this study aimed to investigate whether atherosclerotic plaques in the head and carotid arteries exhibit similar characteristics to those in the coronary arteries within the same individual.

There were three important findings in the current study. First, patients who develop ASCVD exhibit higher stenosis grades in both cervicocephalic plaques and coronary plaques, but no similar trends are shown between the two in terms of other characteristics. Second, we found distinct variations in stenosis, plaque length, plaque volume, and stenosis grades among the culprit plaques, non-culprit plaques, and non-event plaques. Third, among the compositional characteristics of the three different types of plaques, in coronary plaques, the culprit plaque exhibited greater FLPV and FPV than the non-event plaque; in cervicocephalic plaques, the culprit plaque had greater IPHR and LPR than the non-culprit plaque and non-event plaque, whereas the non-culprit plaque had greater FPV than the culprit plaque.

For the ASCVD group, only one morphological characteristic, stenosis grade, was different from the non-event group in the coronary and head and neck artery analyses. We considered that this might be due to the fact that we included all the plaques obtained from the patients in the analysis. For patients who develop the ASCVD, the culprit plaques have certain pathological characteristics that are distinct from those seen in stable lesions, such as the non-culprit plaque and non-event plaque (21). However, due to our comprehensive analysis of all the plaques obtained from the patients, this difference may be covered up. So, we further conducted an analysis focusing on three different types of plaque: culprit plaque, non-culprit plaque, and non-event plaque.

In analyzing the quantitative characteristics of the three different types of plaque, we found that culprit plaques, non-culprit plaques, and non-event plaques differ in stenosis, stenosis grades, plaque length, and plaque volume. In coronary plaques, the culprit plaque had greater stenosis and higher stenosis grades than the non-event plaque; this is similar to the results of previous studies (7). But these differences were not observed in the culprit plaque and the non-culprit plaque. We consider that, for the same patient, adverse plaque features are the main cause of culprit lesion appearance (22). In the analysis of cervicocephalic plaques, we found that although the culprit plaque had greater stenosis and higher stenosis grades than the non-culprit plaque and the non-event plaque, the median culprit plaque stenosis was 0.33, which is a mild stenosis. Since MRI has a good soft tissue contrast and each patient underwent enhanced examination (14), further analysis found that more than half of the culprit plaques had IPH, and all culprit plaques had enhancement, with more than half displaying a grade 2 degree of enhancement. A previous study showed that culprit plaque enhancement or the enhancement ratio was related to stroke (15). Yang et al. (6) found that in cases of low-grade stenosis (<50%) plaques, the culprit plaque demonstrated a higher degree of contrast enhancement and the presence of IPH, but contrast enhancement was associated with the occurrence of cerebrovascular events, while IPH was not. Our findings are similar to previous studies.

Significant differences were identified in both plaque volume and plaque length in the analysis. However, in the cervicocephalic plaque, we found that the non-culprit plaque had the greatest plaque length and plaque volume. We considered that this was relevant to the fact that we included all cervicocephalic plaques of the patients for analysis; the plaque volume and plaque length varied considerably from vessel to vessel, and some long segments and large plaques were included in the non-culprit plaque. Our findings on the comparison of the culprit plaque against non-culprit plaque and non-event plaque showed significantly higher plaque volume and plaque length. This is similar to the findings of the study conducted by Tesche et al. (17).

Several previous studies also demonstrated that culprit lesions had higher non-calcified plaque volume, large fibro-fatty (analyzing in coronary plaques only), and necrotic core volume (17, 23, 24). We found that the culprit plaque in coronary artery had larger FLPV and FPV than the non-event plaque, but the corresponding FLPR and FPR were not significantly different, so the difference in volume may be due to the culprit plaque having a larger volume. However, a larger FPR can be observed in the plaques of the ASCVD group, which means that compared with the non-event group, the overall plaques of ASCVD patients have richer fibrous components. In the cervicocephalic plaque analysis, the culprit plaque had a greater LPR than the non-culprit or non-event plaque, although there was no difference observed in LPV. A larger LPR means a greater proportion of the volume of necrotic core in the plaque; the larger necrotic core also generally means a greater plaque vulnerability (25). One possible reason could be a larger lipid-rich necrotic core (LRNC) triggering increased macrophage activity, which in turn leads to more matrix metalloproteinase secretion (26). This heightened vulnerability may result in plaque rupture and embolism, even without significant narrowing of the artery.

There are some limitations in this study. First, due to the retrospective nature of the study, we cannot completely avoid selection bias, which may limit the application of our results to the larger population. To mitigate this, we used strict inclusion and exclusion criteria to minimize this potential bias. Second, being a single-center study, the number of patients in which CCTA and HR-VWI scans were performed together was small, and the number of patients with co-occurring cardiovascular and cerebrovascular ischemic events was too small, leading to potentially poorer stability of the data. Third, the data were derived from two different images, HR-VWI and CCTA, and the differences in the sensitivity of the two examinations to different plaque components may also potentially affect the analysis of plaque components. Fourth, because there is no pathological confirmation, the composition of coronary plaques and cervicocephalic arterial plaques obtained on the basis of subject imaging is largely based on the conclusions of previous studies, with some possible discrepancies from the actual situation. Fifth, although this study used a mixed-effects model to improve the statistical accuracy, but due to the relatively small sample size, no adjustments were made to account for covariates. Finally, our results are currently a preliminary exploration of whether there are similar trends in the characteristics of coronary and head and neck arterial plaque in patients with or without ASCVD event, and the clinical application of the results is less explored. In the future, we will continue to conduct in-depth research to explore the clinical guidance of the relationship between coronary arteries and head and carotid artery plaques.

## 5 Conclusion

This study conducted an initial exploration into whether the characteristics of cervicocephalic and coronary plaques obtained through different imaging methods are similar within the same patient. Our study revealed that, for the same patient, there is a consistent pattern of change in plaque characteristics between the cervicocephalic and coronary arteries.

## Data Availability

The original contributions presented in the study are included in the article/Supplementary Material; further inquiries can be directed to the corresponding author.
