# Supplementary material for: The value of coordinated analysis of multimodal atherosclerotic plaque imaging in the assessment of cardiovascular and cerebrovascular events
Source: Front Cardiovasc Med. 2024 Jan 25;11:1320222. doi: 10.3389/fcvm.2024.1320222 (PMC10850297; doi:10.3389/fcvm.2024.1320222)
Supplement: Supplementary file 1 [file Table1.docx]

Supplementary Material

# Materials and methods

## Study population

The diagnosis criteria of each vascular risk factor were listed as follows

(1) Hypertension was defined as systolic blood pressure ≥140mmHg and/or diastolic blood pressure ≥90mmHg when clinical blood pressure was measured three times on different days without the use of antihypertensive drugs according to the Chinese Guidelines for the Prevention and Treatment of Hypertension (2018 Revision)；or a history of hypertension treated with antihypertensive drugs.

(2) Diabetes mellitus was defined as typical diabetic symptoms polydipsia, polyuria, unexplained weight loss and random blood glucose test ≥11.1mmol/L or fasting blood glucose test ≥7.0mmol/L or Oral Glucose Tolerance Test (OGTT) 2h blood sugar ≥11.1mmol/L, or hemoglobin A one-c (HbA1c) ≥6.5%, according to the Chinese Guidelines for the Prevention and Treatment of diabetes mellitus type 2 (2020 edition); or a history of diabetes currently using hypoglycemic drugs.

(3) Dyslipidemia according to Guidelines for prevention and treatment of dyslipidemia in Chinese adults (2016 Revision) was defined as serum triglyceride ≥ 1.70mmol/L, or total cholesterol ≥ 5.20mmol/L, or LDL-C ≥ 3.40mmol/L, or HDL-C < 1.00mmol/L; or a history of Dyslipidemia currently taking lipid-lowering drugs.

(4) Smoking was defined as follows: the research subject who stated “they have never smoked” was considered as a never smoker. The research subject who indicated “smoking every day or smoking but not every day” was considered as current smoker. The research subject who indicated “previously smoked but not now” was considered as a former smoker. The smoking rate is the proportion of current and former smokers in the study subjects.

(5) Drinking was defined as women having more than 20g alcohol per day, men intaking more than 40g alcohol per day, and the continuous drinking time is greater than 5 years, or as people intaking more than 80g per day over a two-week period.

## CCTA Imaging parameters

***Contrast:*** 38 ~ 55 ml of ioformol with a concentration of 350mgI/ml was injected into the patient's body through the right anterior cubital vein. The reference standard for the injection rate of contrast agent was: 3.5ml/s for 70kV, 3.7ml/s for 80kV, 4.0ml/s for 90kV, 4.3ml/s for 100kV, 4.6ml/s for 110kV and 5.0ml/s for 120kV. The maintenance time is 11s. Then 0.90% 50 mL sodium chloride was injected at the same flow rate.

***Scanner program:*** Calcification score scans were performed to calculate the Agatston score of the coronary arteries. Then the start software intelligently triggers the scan with the trigger point set at the central of the scanning field in descending aorta, When the CT value reaches the trigger threshold with180 HU, voice command informed the patient to hold his breath, and the scan is triggered after a delay of 5-6s. The system automatically reconstructs the optimal images both in diastolic and systolic period within a cardiac cycle after the scan is completed. When the coronary artery automatically reconstructed images appear artifacts, the appropriate phase can be manually selected for reconstruction, and the position and size of the reconstruction window should be adjusted if necessary to reduce the respiratory movement or folding artifacts. The final scanning and reconstruction are completed, all the original data is transferred to the post-processing workstation (syngo.via) for image post-processing and analysis. multi-planar reconstruction (MPR), curve planar reconstruction (CPR) and volume rendering (VR) were carried out。

***Scan parameters*：**The tube voltage adopts CARE kV (automatically selected according to the body mass index, the range is 70-120 kV), the tube current adopts the CARE DOSE 4D automatic adjustment mode, the rack rotation time for 0.25s, the collimation width for 160×0.6mm, the slice thickness for 0.75mm, and the slice interval for 0.5mm

# Supplementary Tables

**Table S1.** MR Imaging Parameters

|  | T2WI | T1WI | DWI | T2-FLAIR | 3D-TOF-MRA | 3D-T1WI-SPACE | T2WI-SPACE |
| --- | --- | --- | --- | --- | --- | --- | --- |
| TR/TE | 4200/94ms | 1800/33ms | 3000/58ms | 8000/83ms | 900/16ms | 900/16ms | 1200/122ms |
| FOV | 220×220mm2 | 220×192mm2 | 220×220mm2 | 213×220mm2 | 238×169mm2 | 238×169mm2 | 180×180mm2 |
| Matrix | 320×320 | 196×320 | 190×190 | 217×320 | 317×384 | 318×448 | 248×256 |
| PED | Right to Left | Right to Left | Ventral to Dorsal | Right to Left | Right to Left | Ventral to Dorsal | Ventral to Dorsal |
| Slice thickness | 5.0mm | 5.0mm | 5.0mm | 5.0mm | 0.8mm | 0.53mm | 0.7mm |
| Slice interval | 1.5mm | 1.5mm | 1.5mm | 1.5mm | \ | \ | \ |
| Number of layers | 22 | 22 | 22 | 22 | 336 | 240 | 72 |
| Flip angle | 150° | 125° | 180° | 180° | \ | \ | \ |
| NEX | 1 | 2 | 1 | 1 | \ | \ | \ |
| TA | 1min20s | 1min44s | 1min6s | 1min52s | 8min42s | 8min6s | 5min 7s |
| Fat-suppression | \ | \ | Fat-Sat | Fat-Sat | \ | Fat-Sat | \ |
| Turbo factor | \ | \ | \ | \ | 52 | 52 | 56 |
| Acquisition factor | \ | \ | \ | \ | 2 | 2 | 2 |
| Bandwidth | \ | \ | \ | \ | 186HZ/Px | 446HZ/Px | 514 HZ/Px |

TR: response time. TE: echo time. FOV: field of view. NEX: number of excitations. TA: acquisition time. PED: phase encoding direction

**Table S2.** Enhanced characteristics in HR-VWI between ASCVD group and non-event group.

|  |  | **All-patients** | **Non-event Group** | **ASCVD Group** |  |
| --- | --- | --- | --- | --- | --- |
| **Variables** |  | **N=233** | **N=98** | **N=135** | **p** |
| Enhancement NO. (%) |  | 160 (68.7) | 42 (42.9) | 105 (77.8) | 0.006 |
| Enhancement Grade NO. (%) | 0 | 72(30.9) | 42 (42.9) | 31 (23.0) | 0.001 |
|  | 1 | 127 (54.5) | 49 (50.0) | 78(57.8) |  |
|  | 2 | 34 (14.6) | 7 (7.1) | 27(20.0) |  |

**Table S3.** Enhanced characteristics in HR-VWI between ASCVD group and non-event group.

| **Variable** |  | **Overall** | **Non-event plaque** | **Non-culprit plaque** | **Culprit plaque** | **p** |
| --- | --- | --- | --- | --- | --- | --- |
|  |  | **n=233** | **n=98** | **n=111** | **n=24** | **value** |
| Enhancement NO. (%) |  | 160 (68.7) | 56 (57.1) | 81(78.3) | 24(100) | 0.001*# |
| Enhancement Grade NO. (%) | 0 | 72 (30.9) | 42 (42.9) | 30 (27.0) | 0 (0.0) | <0.001*# |
|  | 1 | 127 (54.5) | 49 (50.0) | 67 (60.4) | 11 (45.8) |  |
|  | 2 | 34 (14.6) | 7 (7.1) | 14 (12.6) | 13 (54.2) |  |

* Significant difference between culprit plaque and non-culprit plaque.

# Significant difference between culprit plaque and non-event plaque.
